# Health and healthcare equity within the Canadian cancer care sector: a rapid scoping review

**DOI:** 10.1186/s12939-023-01829-2

**Published:** 2023-01-28

**Authors:** Leah K. Lambert, Tara C. Horrill, Scott M. Beck, Amber Bourgeois, Annette J. Browne, Shannon Cheng, A. Fuchsia Howard, Jagbir Kaur, Michael McKenzie, Kelli I. Stajduhar, Sally Thorne

**Affiliations:** 1 Present Address: Nursing and Allied Health Research and Knowledge Translation, BC Cancer, Suite 500, 686 West Broadway, Vancouver, BC V5Z 1G1 Canada; 2 School of Nursing, University of British Columbia, Vancouver, Canada; 3 College of Nursing, University of Manitoba, Winnipeg, Canada; 4 School of Nursing, University of Victoria, Victoria, Canada; 5 Library Services, BC Cancer, Vancouver, Canada; 6 Radiation Therapy Program, BC Cancer, Vancouver, Canada; 7 Division of Radiation Oncology and Developmental Radiotherapeutics, University of British Columbia, Vancouver, Canada

**Keywords:** Health equity, Healthcare accessibility, Cancer, Oncology, Health systems research, Health services research, Marginalized populations, Scoping review

## Abstract

**Background:**

Despite a publicly-funded healthcare system, alarming cancer-related health and healthcare inequities persist in Canada. However, it remains unclear how equity is being understood and taken up within the Canadian cancer context. Our objective was to identify how health and healthcare equity are being discussed as goals or aims within the cancer care sector in Canada.

**Methods:**

A rapid scoping review was conducted; five biomedical databases, 30 multidisciplinary websites, and Google were searched. We included English-language documents published between 2008 and 2021 that discussed health or healthcare equity in the Canadian cancer context.

**Results:**

Of 3860 identified documents, 83 were included for full-text analysis. The prevalence of published and grey equity-oriented literature has increased over time (2008-2014 [*n* = 20]; 2015-2021 [*n* = 62]). Only 25% of documents (*n* = 21) included a definition of health equity. Concepts such as inequity, inequality and disparity were frequently used interchangeably, resulting in conceptual muddling. Only 43% of documents (*n* = 36) included an explicit health equity goal. Although a suite of actions were described across the cancer control continuum to address equity goals, most were framed as *recommendations* rather than direct interventions.

**Conclusion:**

Health and healthcare equity is a growing priority in the cancer care sector; however, conceptual clarity is needed to guide the development of robust equity goals, and the development of sustainable, measurable actions that redress inequities across the cancer control continuum. If we are to advance health and healthcare equity in the cancer care sector, a coordinated and integrated approach will be required to enact transformative and meaningful change.

**Supplementary Information:**

The online version contains supplementary material available at 10.1186/s12939-023-01829-2.

## Introduction

Health equity and equitable access to healthcare are global concerns, made ever more visible during the COVID-19 pandemic. Health equity can be understood as the absence of avoidable or remediable differences in health, both among and between groups of people, and as all people having a fair opportunity to be as healthy as possible [1, 2]. In 2008, the World Health Organization’s (WHO) Commission on the Social Determinants of Health published a landmark report declaring that health inequities were “killing people on a grand scale” ([3] (p1)). This is particularly evident within the cancer care sector. In Canada, there are alarming inequities across the cancer care continuum, resulting from a constellation of socioeconomic, geographic, political, and historical factors, and disproportionately impacting underserved segments of the population. This includes those who experience often-intersecting impacts of racism, stigma, discrimination, poverty or unstable housing, mental health and substance use challenges, and/or disabilities [4, 5]. Research suggests that groups disproportionately impacted by health and social inequities are significantly more likely to be under-represented in cancer control programs, diagnosed with preventable cancers, diagnosed with cancers at advanced stages, receive inadequate cancer treatment, and die from typically curable or treatable cancers [4, 6–13].

As a result, there are growing calls to prioritize health equity and address cancer-related inequities within and outside of the cancer care sector [14, 15]. As an influential intermediate determinant of health, health systems can play an important role in mediating health inequities by taking direct action toward mitigating the impacts of social determinants of health, transforming organizational culture of healthcare, and through intersectoral collaboration [3, 16–18]. Individual healthcare organizations could also play an essential role in addressing inequities at the point of care and through organization-specific strategies aimed at closing the health equity gap, including making equity a strategic priority, partnering with community organizations, and developing organizational structures to support the delivery of equity-oriented care [18, 19]. As a sub-component of the healthcare system, the cancer care sector is increasingly recognized as a critical site for health equity interventions due to the heterogeneous distribution of cancer risk, outcomes, and mortality among populations disadvantaged on the basis of health and social inequities, and the structural factors that generate and perpetuate disparities [20]. Yet, despite increasing attention to health equity and growing calls for healthcare equity to be a priority within the cancer care sector in Canada, it is unclear how health equity is being understood and taken up in this context. In particular, conceptualizations of health equity are widely variable, and although cancer organizations are beginning to foreground policies and strategic plans in health equity and social determinants of health rhetoric, it is not clear whether this is translating into meaningful action [14].

### The Canadian context

The cancer sector provides a wide range of services to individuals, families, and communities, with cancer care often conceptualized as a pathway or continuum extending from cancer prevention and screening, diagnosis, treatment, surveillance and survivorship, and end-of-life care and encompasses clinical care, research, and education [20]. Within Canada, cancer services are publicly funded by both federal and provincial/territorial governments, organized provincially, delivered regionally, and free to access at the point of care. Cancer service organization and delivery are influenced by national organizations (i.e., governance, non-profit and community-based) such as the Canadian Partnership Against Cancer, the Canadian Cancer Society, the Canadian Cancer Research Alliance, and the Canadian Association of Provincial Cancer Agencies.

### Aims

The aim of this review was to explore the peer-reviewed and grey literature to document how health and healthcare equity are being discussed as goals or aims and/or operationalized within the Canadian cancer care sector. This review was conducted in the initial phase of a one-year funded project which aimed to develop research partnerships, facilitate knowledge exchange, and identify recommendations for promoting equity within the cancer care sector. Our review team included researchers with internationally-recognized expertise in health equity and cancer research, as well as oncology clinicians, as described in our protocol [21].

### Theoretical perspectives

This review was guided by critical social justice perspectives, and the central concepts of health equity and social determinants of health, which emphasize systemic and social factors shaping health. Understanding health as a basic human right, critical social justice theorizes health inequities to be rooted in power imbalances and embedded in historical, economic, and political dimensions [22]. Rather than focusing exclusively on health*care access*, a critical social justice perspective focuses on health *outcomes* and access to *resources for health* at the group or collective level [23]. The WHO’s definition of health inequities as “health differences that are socially produced, systematic in their distribution across the population, and unfair” also appeals to ethical norms and human rights by contending that poor health “profoundly compromises freedom” ([16] (p12)).

**Fig. 1:**
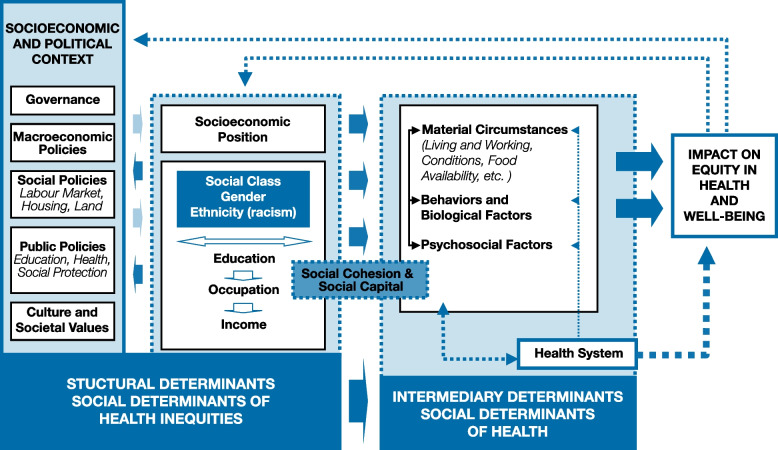
Commission on Social Determinants of Health Conceptual Framework [16]

As systematic differences in health that are both avoidable and unfair, health inequities are created and maintained by social determinants of health, which are, in turn, shaped by structural forces, including social values and contexts, economics, politics, and public policy as depicted in Fig. 1 [16]. Notably, this social determinants of health framework conceptualizes the *health system itself* as an influential determinant of health [16]. This informed the focus of this review in understanding how provincial and national cancer organizations envision and/or address health equity at the health systems level.

## Methods

Given our broad aim of exploring and mapping conceptualizations of health equity within the Canadian cancer care sector, a scoping review methodology was deemed most appropriate. Methods for this scoping review were based on the work of Arksey and O’Malley [24] and Levac and colleagues [25], and are reported in more detail in our protocol [21]. This review was conducted in six iterative stages, expanded upon below. We also drew on the WHO’s “Rapid Reviews to Strengthen Health Policy and Systems” [26]. The rapid review approach was employed as a result of the project timeline and resource limitations. In addition, this review took place within the broader context of a cancer care system in which health equity was identified as a priority; to be responsive and capitalize on this opportunity to advance a health equity agenda, inform policy recommendations, and develop research priorities, the rapid review approach was deemed most appropriate. Our review is reported using the Preferred Reporting Items for Systematic Reviews and Meta-Analyses extension for scoping reviews (PRISMA-ScR) guidelines [27].

### Stage 1: identification of research question

We designed this scoping review to answer the specific research question: *How is health and healthcare equity conceptualized and discussed as a goal or aim within the Canadian cancer care sector?* Key concepts within our research question include health equity, healthcare equity, and the cancer care sector (Table 1).

**Table 1:** Definition of key concepts

Concept	Definition
**Health equity**	The absence of avoidable or remediable differences in health among and between groups of people, ensuring that all people have full access to opportunities that enable them to lead healthy lives, and taking into account social, political, and economic influences [2, 3].
**Healthcare equity**	The absence of avoidable or remediable differences in healthcare access among and between groups of people; taking into account geographic, economic, organizational, sociocultural, and relational influences on healthcare access; and the design and delivery of healthcare services [19, 28–31].
**Cancer care sector**	Health services policy, planning, and delivery with the goal and/or mandate of controlling cancer including: primary prevention, screening, diagnostic services, treatment, surveillance, survivorship care, end-of-life care, and research.

### Stage 2: identification of relevant studies

We identified published and grey (i.e., unpublished) literature from three main sources: (1) five biomedical databases (Ovid MEDLINE(R), Ovid Embase, Ovid EBM Reviews, EBSCO CINAHL, and EBSCO APA PsycInfo); (2) 30 public health and multidisciplinary websites and databases; and (3) the broader Internet. Database searches were completed by a medical reference librarian (SC) with input from the principal investigator (LKL) and postdoctoral fellow (TCH). The initial search strategy was peer-reviewed by a health librarian external to the research team (Prubjot Gill), using the Peer Review of Electronic Search Strategies guideline and checklist [32]. A full description of the search strategy is included in Additional file 1. Published and grey literature were screened using the same inclusion and exclusion criteria.

### Stage 3: study selection

We used a two-step process for study selection. To accommodate the rapid nature of this review and screen a large number of documents in a short timeframe, multiple team members screened documents using the inclusion/exclusion criteria (Table 2):

1. A team of reviewers screened titles and abstracts of documents for eligibility against the inclusion/exclusion criteria. Each abstract was reviewed by a single screener, with 10% of abstracts verified for inclusion or exclusion by a second reviewer [26].
2. To determine eligibility for inclusion, the full text of articles included in Step 1 were reviewed using the same process described above.

**Table 2:** Inclusion and exclusion criteria

Criterion	Inclusion	Exclusion
**Language**	English	All other
**Country**	Canada or substantial Canadian focus	All other
**Date**	2008 – 2021	All other
**Document type**	Peer-reviewed publications (research, discussion papers, theoretical papers, reviews), organizational documents, policies, strategic plans, reports, position statements^a^	Theses/dissertations, clinical practice guidelines, conference proceedings, slide presentations, news stories
**Health equity concept**	Use of terms ‘health equity’, ‘healthcare equity’, ‘health inequity’, ‘healthcare inequity’, or their variations (inequality, disparity)	Do not use specified terms, refer to determinants of health without reference to health equity (or variant term)
**Health equity goal or action**	Discusses a health equity (or related term) goal or aim explicitly, or discusses health equity actions that imply a health equity goal or aim	Does not discuss a health equity (or related term) goal or aim explicitly or implicitly
**Cancer care sector**	Focus on one or more points along the cancer continuum: prevention and screening, diagnosis, treatment, surveillance, survivorship care, end-of-life care	Focus is outside of the cancer continuum or external to the cancer care sector
**Health systems perspective**	Focus on health equity from a health systems perspective: health financing, policy, planning, structuring, management, healthcare access, workforce and human resources, service delivery, leadership and governance	Focus is not on the health system; e.g., specific clinical care (e.g., applying ice during chemotherapy), cancer treatments, clinical trials

^a^For reports, strategic plans (etc.) with multiple or yearly editions, we only included the most recent version in our analysis

**Fig. 2:**
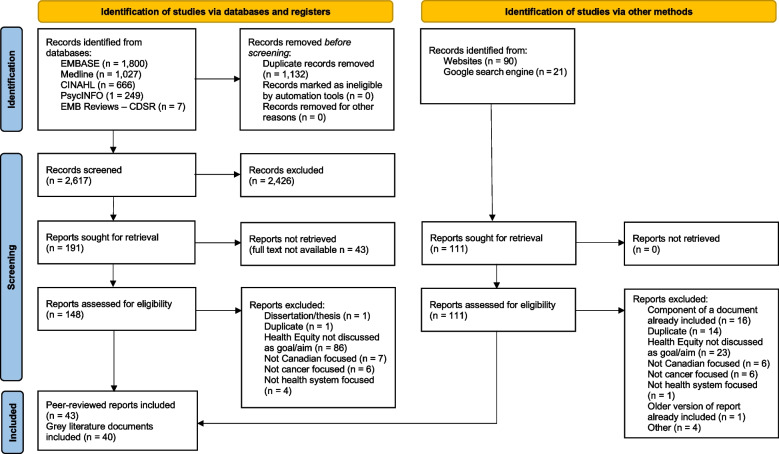
PRISMA Scoping Review Flow Diagram

Specifically, published or unpublished research, policy documents or strategic plans that discussed health equity within the context of cancer care, or those that had a stated goal, aim, or mission that focused on health equity from a health systems perspective were included. Articles or documents published in English, or those with an associated English version, were included. Articles or documents published prior to 2008 were excluded, as we envisioned mapping current rather than historical conceptualizations of health equity. Moreover, the publication of the WHO’s Commission on the Social Determinants of Health report in 2008 was arguably the start of significant shifts in understanding of the concepts of health equity and social determinants of health, and increased attention to these concepts in research, policy, and practice. Reviewers met weekly during the selection process to discuss and clarify decisions in abstract and full text screening. We used Covidence software (www.covidence.org) to manage the study selection process, reported in Fig. 2 according to the PRISMA extension for scoping reviews checklist [27]. See Additional file 2 for a complete list of included documents.

### Stage 4: charting (extracting) the data

Data extraction was completed in a standardized format by one reviewer per document using Covidence for peer-reviewed literature and Excel for grey literature, with 10% of data verified by a second reviewer [26]. We pilot tested our data extraction form with a sample of five documents, and met as a team to clarify elements for extraction (Additional file 3). To ensure a consistent approach throughout data extraction, we met weekly or bi-weekly.

### Stage 5: collating, summarizing and reporting the results

In the final stage, we engaged in an iterative data analysis process as a team to map the literature on health equity with the cancer care sector. We conducted a content analysis to analyze and summarize the content of included documents with respect to conceptualizations of health equity, discussion of health and/or healthcare equity goals, and reported actions to support health and healthcare equity goals. Findings are reported as a narrative summary [25].

### Stage 6: consultation

The final stage in the scoping review framework included consultation and engagement with key stakeholders regarding the study findings and potential implications [24, 25]. This scoping review was part of a larger project that brought together healthcare providers, researchers, leaders in health policy and service delivery, and knowledge users to discuss health and healthcare equity as it relates to cancer care and to develop a research team interested in applying evidence-informed knowledge to pursuing new research to promote equity in the cancer care system. We engaged these key stakeholders through a series of three virtual meetings, led by the first author (LKL), in which we presented preliminary findings, sought feedback, and engaged in facilitated dialogue on how current cancer care practices, policies, and systems contribute to inequities. These sessions helped to interpret review findings and identify important implications for policy, practice, and future research.

## Results

**Fig. 3:**
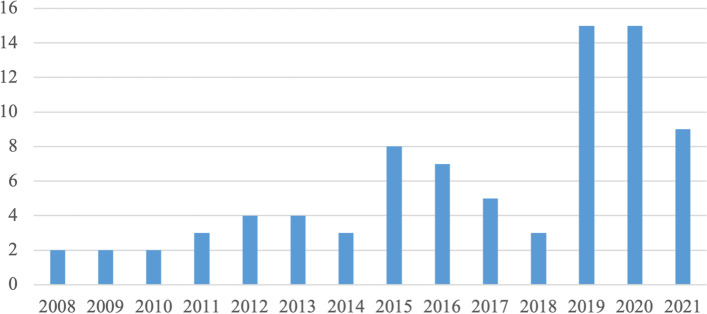
Number of Papers Included in the Review by Publishing Year*

A total of 83 documents were included in this scoping review (Fig. 2). The majority of included documents were original research (*n* = 24), discussion or commentary papers (*n* = 17), and reports (*n* = 15) (Table 3). There was an average of 2.8 documents per year published between the years of 2008 and 2014 (range: 2-4), which increased significantly to 8.3 documents per year between 2015 and 2021 (range: 2-15) (Fig. 3).

**Table 3:** Types of documents included

Published Article Type	Number of Articles
Original research	24
Discussion/Commentary	17
Review	2
Strategic plan	8
Report	15
Policy document	4
Webpage	3
Other^a^	10

^a^Toolkit, environmental scan, casebook, framework, educational module, guideline, compendium, quality improvement report

### Conceptualizations of health equity

As a first step, we were interested in identifying whether or how documents provided a clear definition of health or healthcare equity or related terms, the types of terms used, and the ways in which the terms were defined. A range of terms was used when referring to health or healthcare equity, including *equity* or *equitable, inequity, disparity, equality, inequality,* and *underserviced* or *underserved*. Terms were often used interchangeably [33–36], and most often, this was seen in relation to *equity/inequity* and *equality/inequality*. For instance, equality of access in one paper was defined as services in proportion to need [34], which is instead more reflective of the concept of equity.^1^

Only one-quarter of included documents (*n* = 21) provided an explicit definition of the terms used. Several documents contained definitions of health equity that addressed key aspects of the WHO’s definition of health equity, including the absence of differences in health at the *group level* that are *socially produced,* and that are both *avoidable* and *unfair* [37, 38]. However, other definitions were confusing, with multiple concepts used within the same definition (e.g., equity, disparity, inequity), or problematic when inequities and disparities were alternatively referred to as differences and variabilities, which obscures the systematic, avoidable, and unfair nature of inequities [39]. In some documents, the concept of equity was equated with healthcare equity, wherein authors discussed health equity as achieved when there is equitable access to healthcare [40–42].

As a component of understanding how health equity was conceptualized, we were interested in whether documents acknowledged or discussed the causes of health inequities. Notably, only 21 documents (25%) included a definition of health equity and 76 documents (92%) discussed potential or known causes of health inequities, either broadly or specifically in relation to cancer inequities. Using the WHO’s Social Determinants of Health (Fig. 1), we analyzed how the causes of health inequities were discussed using four categories: (1) social determinants of health, (2) behavioral/biological determinants of health, (3) social determinants of health inequities, or (4) structural determinants of health inequities. *Social determinants of health* were most commonly discussed (*n* = 64 documents), with geography and health systems factors identified as key contributors to health inequities. Although sometimes identified simply as geographical location, many documents discussed how geography often impacts the types of services and providers available (e.g., specialty cancer care is concentrated in urban areas) and associated transportation challenges. Health system factors included complexity of the design, organization, and delivery of care (e.g., ‘siloed’ and disconnected systems of care [43]; models of care (e.g., absence of patient-centered or culturally appropriate models of care [33, 44]); and healthcare provider interactions and patients’ negative experiences of care (e.g., differential or discriminatory treatment by healthcare providers [5, 40]).

*Social determinants of health inequities* (*n* = 44 documents) include factors that determine socioeconomic positioning and relative degree of advantage. Income was frequently discussed as a determinant of health inequity, either alone or in combination with other factors such as education [45–48]. Stigma, discrimination and racism were acknowledged in some documents [34, 36, 43, 49]; however, more often factors such as ‘race’, ethnicity, immigrant status, language, religion, or whether someone was foreign-born or of a visible minority were discussed [4, 41, 50, 51]. *Structural determinants of health inequities* are broad, contextual factors that, although difficult to measure directly, exert powerful influences on societies [16]. Structural determinants of health inequities (*n* = 30 documents) discussed in the literature included the distribution of power [38, 52], systemic racism [53, 54], health policy [37], and colonialism and its effects [55–59]. In contrast, few documents (*n* = 10) discussed behavioral (e.g., personal health beliefs [60]) or biological (e.g., genetics [61]; age and sex [62]) factors as contributing to inequities.

### Reported health and healthcare equity goals

Less than half of the included documents contained an explicit health or healthcare equity goal (*n* = 36). Explicit equity goals were more common in the grey literature (*n* = 24) than peer-reviewed documents (*n* = 12). Both health and healthcare equity goals were discussed, and ranged from broader, more general goals to those that were more focused and specific [33, 54, 63, 64]. General health equity goals were primarily related to improving health equity broadly or ensuring equitable cancer outcomes. In contrast, more focused and specific goals aimed to reduce inequities in cancer incidence, survival, or risk factors. Although healthcare equity goals were described in both broad and specific ways, they focused almost exclusively on equitable access to care. A small set of documents (*n* = 5) included both health *and* healthcare equity goals; for example, “to ensure equitable, person-centered cancer control across the care trajectory, with the long-range goals being that fewer Canadians develop cancer, more people survive cancer, and those with cancer have a better quality of life” ([54] (p4)). Of the remaining documents that did not discuss an explicit goal (*n* = 47), equity goals or aims could often be implied or inferred based on the types of actions, recommendations, or next steps discussed and pertained mainly to a need for equitable access to oncology care.

Only 11 documents described how progress on the health or healthcare equity goal would or had been measured or monitored. For example, the 2016 Manitoba Cancer Plan (a plan guiding the design and delivery of cancer services in the Canadian province of Manitoba) identifies a goal to “improve care for underserved populations” and “ensure equitable access to cancer services and care for all Manitobans” [65]. This will be monitored through performance indicators, including the number of participants in underserved populations supported each year and the percentage of underserved populations meeting cancer service targets (e.g., wait times, treatment according to practice guidelines) relative to other Manitobans [65].

### Health and healthcare equity actions in support of goals

**Fig. 4:**
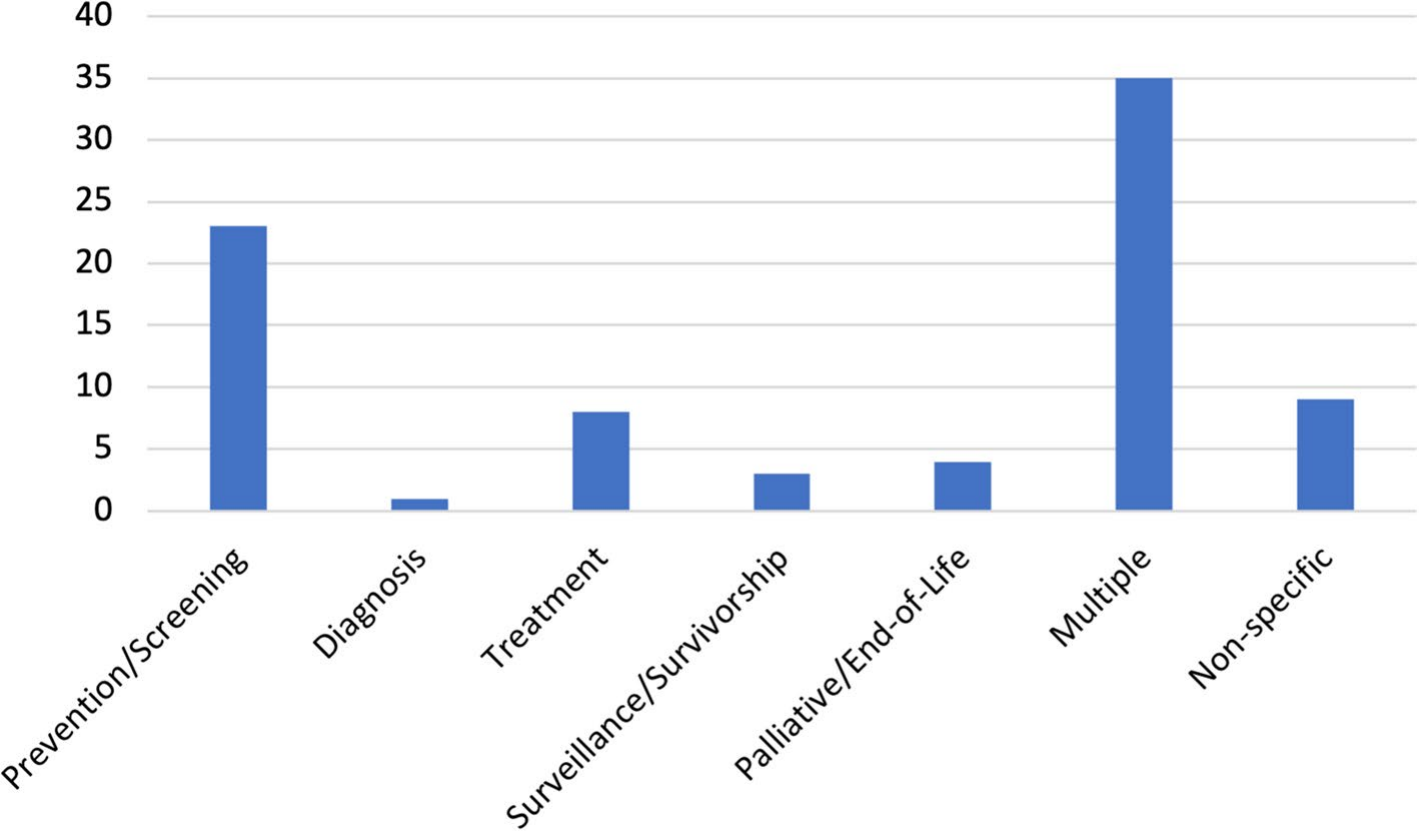
Number of Documents that Identified Actions per Point on the Cancer Continuum

A wide range of actions (interventions, policies, strategies, recommendations) to address stated health and healthcare equity goals were described within the included documents. About two thirds of documents described goals or actions that targeted a range of population groups (Table 4), with many being focused at the national (*n* = 28) or provincial level (*n* = 25 total; 15 Ontario, 4 Manitoba, 3 Alberta, 2 British Columbia, 1 Nova Scotia), with fewer focused at the regional (*n* = 9) and organizational level (*n* = 3). Actions were often directed at multiple points on the cancer continuum simultaneously (*n* = 35), with those targeting a single point on the cancer continuum mainly focused on health promotion, prevention, and/or screening (*n* = 24) (Fig. 4).

**Table 4:** Target group or population of equity goal or action

Target Group or Population	# of Documents
Point on cancer continuum	3
Health condition	
Physical illness	3
Mental illness	3
Biology	
Age	4
Sex	5
Type of cancer	5
Social location	
Sexual orientation	2
Disability	2
Immigration status	5
Ethnocultural identity	8
Gender identity	8
Socioeconomic status	12
Indigenous identity	20
Geographical location (urban, rural, remote or specific geographic region)	13

Given our interest in the health system as an intermediate determinant of health, and how health equity is being discussed or addressed within the cancer care sector specifically, we extracted and categorized health equity actions as *health systems improvements*, *policy and planning changes*, *point of care improvements*, *research*, or *other*. The majority of documents described actions aimed at health system improvements (*n* = 52). *Health systems improvements* were aimed at improving coordination or integration of care [13, 43, 44, 53, 59, 64, 66, 67], increasing the number of Indigenous healthcare providers and health workforce ‘diversity’ [55, 68–70], and expanding service provision including telehealth and mobile services across the continuum [5, 42, 58, 66, 71–74]. Recommendations for work to improve health systems also included calls for the co-design of cancer services with stakeholders [33, 75, 76], and to re-design cancer services, develop new models of care, or implement existing models of care. Models of care that were highlighted included ‘hub-and-spoke’ models [71, 77], person-centered care models [48, 76], nurse-led care models [36, 76], and models of care focusing on upstream and social determinants of health [13, 38, 68, 78, 79].

Over one third of documents specified actions related to *policy and planning* (*n* = 31). Some actions were broad, for example, calling for policy to address social determinants of health [20, 50, 57, 78, 80]. Funding-specific policy recommendations focused on ensuring equitable access to cancer drugs and treatments [48, 81–83], or funding specific services or interventions to address inequities [60, 66, 67, 80, 83]. Additional documents specified actions related to *point of care improvements* (*n* = 19), including ensuring care is tailored to individual and/or community needs and addresses non-physical needs [34, 70, 84], as well as improving communication with patients (verbal and written) and scheduling more time at appointments [67, 70, 76, 85, 86]. Importantly, additional point of care actions included ensuring care is delivered in ways that mitigate stigma and bias, are anti-racist, culturally safe, trauma-informed, and welcoming and respectful [38, 43, 51, 67, 68, 78, 87]. Actions related to *research* included the need to use existing data and conduct research to better understand existing inequities [4, 33, 41, 62, 68, 75, 79, 88–91], and research that specifically seeks to untangle the distinctive roles of racism, stigma, and social determinants of health on cancer experiences, outcomes, and inequities [5, 52, 57, 78, 89]. It was also clear in the reviewed documents that there are significant data gaps and considerable need for more and better data related to health equity in cancer care, including the need for data sharing policies and national databases [5, 47, 52, 55, 58, 85, 88, 92].

Actions categorized as *other* included a range of strategies, most notably related to education and collaboration. Actions targeting education included the need for healthcare provider education that incorporates an intersectional lens on racism, stigma, and social determinants of health [5, 49, 55, 79, 93], and recommendations specific to the need for education on colonialism and culturally safe care [5, 40, 51, 55, 66, 67, 79, 91, 94]. Specific areas where patient education could be helpful were also noted, including, for example, the need for culturally appropriate and empowering education for women on cervical cancer screening [66] and one-to-one education with patients on the benefits of colorectal cancer screening [70]. Suggested actions related to partnership and collaboration included the need to partner with communities, non-governmental organizations, and other stakeholders to: take intersectoral action on health equity within the cancer care sector, ensure services are designed to meet the needs of those they are serving, and design and conduct research and knowledge transfer and exchange activities [5, 20, 50, 56, 58, 68, 70, 74, 91, 92, 95, 96].

## Discussion

Building on decades of work by scholars and activists, health and healthcare equity is an emerging priority in Canada’s cancer care sector and this growing focus on equity is encouraging. While our review focused specifically on the Canadian context, the increasing interest in health equity in Canada’s cancer sector may be reflective of the growing attention to and stated importance of health equity broadly [15, 97] and within the field of oncology globally [98]. In particular, the work of the American Society of Clinical Oncology (ASCO) has been influential in drawing attention to equity as a key priority for cancer care through its policy and position statements, research and educational initiatives (https://www.asco.org/news-initiatives/current-initiatives/health-equity).

As a mounting priority within the cancer care sector, the growing attention to health equity presents several challenges. In particular, we noted problematic omissions within the literature, including lack of definitions and inconsistent use of the concept of health equity. This is similar to the phenomenon observed within the broader health equity literature, in which the concept of health equity is frequently referred to, without articulating a common understanding of what it means [1, 99]. In this review, our findings highlight how the absence of clear conceptual definitions and/or the inconsistent use of concepts has resulted in conceptual ‘muddling’. Our concern with this is twofold, and is informed by Lett and colleagues’ critique of *health equity tourism* [100]. First, conceptual muddling *pollutes* the health and healthcare equity literature with work that does not correctly articulate social and structural injustices as the root-causes of health and healthcare inequities, and risks conflating health equity with other terms that may sound similar, but which have different objectives and paths to address those respective goals. Second, conceptual muddling *dilutes* the prevalence of thoughtfully constructed, high-quality and theoretically grounded work by those with the necessary health equity expertise required to redress health and healthcare inequities. In other words, poorly or incorrectly characterizing the determinants of health and healthcare inequities in the cancer care sector is potentially harmful. At best, the pollution and dilution of the concepts of health and healthcare equity is the result of incomplete scholarship; at its worst, it has the potential to reinforce notions of individual responsibility over health and illness, to devalue the efforts of scholars and activists, and to perpetuate existing health and healthcare inequities. Considering the potential harms of conceptual muddling, future work to advance health equity in the cancer care sector ought to present carefully and deliberatively considered definitions of key terms.

Redressing health and healthcare inequities not only requires clearly stated goals, but it must also be paired with meaningful action. This review showed that the majority of documents in the Canadian cancer care literature do not explicitly state a health equity goal. The goals outlined most often captured either health equity goals or healthcare equity goals, but rarely both, and in most instances it was not articulated how these goals would be measured or monitored. While individual level efforts of leaders and clinicians are helpful in working towards equitable cancer care for patients and families, the lack of clearly defined health equity goals by researchers, health systems leaders, and policymakers limits the potential for strategic, collaborative goals that can be implemented, measured, and evaluated [1, 18]. What will be essential in redressing health inequities, are explicit health equity goals that work towards collective, system level changes, coupled with intersectoral collaboration and coordination aimed at promoting equity more broadly [18]. A related and concerning observation was how few documents described strategies to measure and/or evaluate progress toward achieving health equity goals, or the impact of proposed actions. Although this finding is likely a function of poorly defined equity goals, the development of conceptual and operational definitions to guide the measurement of health and healthcare equity is essential [99].

Finally, the results of this rapid scoping review revealed that the majority of ‘actions’ presented in the documents we analyzed were described as *proposed* actions or *recommendations*, with very little evidence of actions, strategies, or interventions that had actually been taken or implemented. Although it was promising to see some documents had well-defined, meaningful recommendations at the health systems and policy levels that, if implemented, may improve health equity in the cancer care sector, movement from recommendations to action is needed. The lack of demonstrated progress toward achieving health equity goals may be reflective of equity as an emerging focus within the cancer care sector, but may also be indicative of an entrenched healthcare sector that is resistant to change that may disrupt power structures and long-held ways of doing and being in healthcare [19].

### Limitations

Due to the rapid nature of this scoping review, and the corresponding constraints on time and resources, there are some potential limitations to our search strategy. First, forward and backward citation searches were not conducted. We anticipated that the scholarly databases would cover much of the health equity literature and, thus, prioritized more time for searching the grey literature. Second, relevant publications in disciplines outside of the health sciences may have been missed. With limited access to databases, our search for published literature relied heavily on biomedical databases. Third, despite clearing the browser’s history, cookies, and site data, and limiting our search results by file type and region, our internet-wide advanced Google search may have been biased due to features of Google’s search algorithm. Finally, we were limited to English language publications (with the exception of a limited search of the Quebec Ministry of Health and Social Services/ Ministère de la Santé et des Services Sociaux, described in Additional file 1), which may have excluded relevant content produced by non-English speaking scholars or organizations. This has particular relevance given Canada’s two official languages (i.e., English and French).

## Conclusion

Achieving health and healthcare equity in Canada’s cancer care sector will require the coordinated and integrated efforts of clinicians, researchers, educators, policymakers, and system leaders alike. However, ameliorating health and healthcare inequities in the cancer care sector—and across health systems more broadly—will depend on more than vague strategies and recommendations. This rapid scoping review identified significant areas for improvement, including the need for improved conceptual clarity, the clear articulation of equity goals, and the development of sustainable, meaningful actions that redress inequities across the cancer control continuum. Greater attention to the systematic, avoidable, and unfair nature of cancer-related health and healthcare inequities is essential to closing the equity gaps in Canada’s cancer care sector.

## Supplementary Information

**Additional file 1.** Description of search methods.

**Additional file 2.** Complete list of documents included for analysis.

**Additional file 3.** Variables for data charting.

## Data Availability

Restrictions apply to the availability of the data that support the findings of this review, which were used under license and so are not publicly available.

## Abbreviations

ASCO: American Society of Clinical Oncology
PRISMA-ScR: Preferred Reporting Items for Systematic Reviews and Meta-Analyses extension for scoping reviews
WHO: World Health Organization

## Footnotes

1 Key references have been included as examples to support our analysis but are not an exhaustive list.
