# Supplementary material for: Health and healthcare equity within the Canadian cancer care sector: a rapid scoping review
Source: Int J Equity Health. 2023 Jan 28;22:20. doi: 10.1186/s12939-023-01829-2 (PMC9883825; doi:10.1186/s12939-023-01829-2)
Supplement: Supplementary file 1 — Additional file 1. Description of search methods. [file 12939_2023_1829_MOESM1_ESM.pdf]

ADDITIONAL FILE #1:
Search Methods

We searched five biomedical databases: (1) Ovid MEDLINE(R) and Epub Ahead of Print, In-Process, In-Data-Review & Other Non-Indexed Citations and Daily; (2) Ovid Embase; (3) Ovid EBM Reviews - Cochrane Database of Systematic Reviews; (4) EBSCO CINAHL; and (5) EBSCO APA PsycInfo. We limited the search date to publications from 2008 onwards. This year was chosen in accordance with the key/ seminal report by the World Health Organization (WHO) on the social determinants of health. No other filters (eg. language) were applied. The results were imported to Covidence and screened independently by the review team.

The nature of the topic led us to anticipate more evidence in the form of grey literature than in published literature. For guidance on the grey literature search method, we drew from Grey Matters: A Practical Tool for Searching Health-Related Grey Literature (CADTH, 2019), State-of-the-evidence reviews: advantages and challenges of including grey literature (Benzies et al., 2006) and Searching and synthesising 'grey literature' and 'grey information' in public health: critical reflections on three case studies (Adams et al., 2016).

Between July 19 and August 10, 2021, we searched 30 targeted Canadian- and North American-focused public health and multidisciplinary websites and databases by hand-searching (browsing), using the website's built-in search feature, and by completing site searches using Google's search API (code available at https://github.com/scmlis/cancer-equity).

To extend the breadth of search, we completed an Internet-wide search for grey literature using Google. The grey literature search terms included keywords on equity, cancer, and Canada. To minimize bias, increase search transparency, and to expedite the search process, we used Google's search API to perform targeted site searches. For some websites, browsing and using the website's search feature produced incomplete or broken results. Supplementing this with Google's search API enabled us to capture content that was missed due to website inconsistencies. In cases where inconsistencies could not be resolved, the website administrators were contacted for clarification and content retrieval. In all of these cases, no relevant materials were found.

To search the Quebec Ministry of Health and Social Services/ Ministère de la Santé et des Services Sociaux, we consulted a francophone health librarian (Elena Popova) in case there was French content that could be requested in English. However, there were no relevant results. In total, 19 keyword searches were completed for each of the 15 targeted cancer websites, 4 additional keyword searches in French were completed for Quebec Ministry of Health and Social Services/ Ministère de la Santé et des Services Sociaux, and 38 keyword searches were completed for the non-oncology websites. For health websites that had a different link for their cancer program, we searched both sets of 19 keywords and 38 keywords to be thorough.

The broader Internet-wide search relied on Google's Advanced Search feature to search for cancer and health equity concepts with the file type limited to PDFs and the region set to Canada. To discover relevant results, while recognizing the extensive coverage of the other grey literature searches and the limited timeframe, we followed common practices and included the first 10 pages (or 100 results per search) of each Google search for screening (see Systematic Literature Searching: A Resource Guide - Alberta, Optimizing the Identification of Grey Literature: A Rapid Review). All of the grey literature was exported to an Excel spreadsheet for efficient screening, with the exception of websites that did not have a persistent search link; these searches were saved as a PDF for screening.

Database Search Strategy:

Ovid MEDLINE(R) and Epub Ahead of Print, In-Process, In-Data-Review & Other Non-Indexed Citations and Daily <1946 to July 15, 2021>

1. Health Equity/
2. Health Services Accessibility/
3. Right to health/
4. Healthcare Disparities/
5. Health status disparities/
6. "Social Determinants of Health"/
7. Socioeconomic Factors/
8. ((health* or care) adj4 (Inequal* or unequal* or equal* or disparit* or gap? or oriented or accessibility or inaccessibility)).mp.
9. (equit* or inequit* or "social determinant*" or "right to health*").mp.
10. ((socio* or social) adj2 (factor* or Inequal* or unequal* or equal* or disparit* or gap? or accessibility or inaccessibility)).mp.
11. or/1-10
12. exp Neoplasms/
13. "Early Detection of Cancer"/
14. Cancer Survivors/
15. exp Medical Oncology/
16. Oncology Nursing/
17. Oncology Service, Hospital/
18. Cancer Care Facilities/
19. (neoplas* or cancer* or tumor* or tumour* or carcinoma* or adenocarcinoma* or sarcoma* or leiomyosarcoma* or malignan* or oncolog*).mp.
20. or/12-19
21. Indians, North American/
22. Indigenous Canadians/
23. Inuits/
24. (Canad* or "British Columbia*" or Vancouver* or Alberta* or Calgary or Calgari* or Edmonton* or Saskatchewan* or Manitoba* or Ontari* or Ottawa* or Toronto* or Quebec* or Montreal* or "Nova Scotia*" or "Prince Edward Island*" or Newfoundland* or Labrador* or Nunavut* or NWT or "N.W.T" or "Northwest Territories" or Yukon* or Nunavik or Inuvialuit or Inuit* or Inuk or "First Nation" or "First Nations" or Metis or "Métis").ab,ti,kf,hw.
25. or/21-24
26. 11 and 20 and 25
27. limit 26 to yr="2008 -2021"

Ovid Embase <1974 to 2021 Week 27>

1. health equity/
2. health care access/

3. right to health/
4. health care disparity/
5. "social determinants of health"/
6. socioeconomics/
7. ((health* or care) adj4 (inequal* or unequal* or equal* or disparit* or gap? or oriented or accessibility or inaccessibility)).mp.
8. (equit* or inequit* or "social determinant*" or "right to health").mp.
9. ((socio* or social) adj2 (factor* or inequal* or unequal* or equal* or disparit* or gap? or accessibility or inaccessibility)).mp.
10. or/1-9
11. exp neoplasm/
12. early cancer diagnosis/
13. cancer survivor/
14. oncology/
15. psycho-oncology/
16. radiation oncology/
17. surgical oncology/
18. oncology nursing/
19. cancer center/
20. (neoplas* or cancer* or tumor* or tumour* or carcinoma* or adenocarcinoma* or sarcoma* or leiomyosarcoma* or malignan* or oncolog*).mp. [mp=title, abstract, heading word, drug trade name, original title, device manufacturer, drug manufacturer, device trade name, keyword, floating subheading word, candidate term word]
21. or/11-20
22. american indian/
23. canadian aboriginal/
24. first nation/
25. metis/
26. inuit/
27. eskimo/
28. Eskimo-Aleut people/
29. (Canad* or "British Columbia*" or Vancouver* or Alberta* or Calgary or Calgari* or Edmonton* or Saskatchewan* or Manitoba* or Ontari* or Ottawa* or Toronto* or Quebec* or Montreal* or "Nova Scotia*" or "Prince Edward Island*" or Newfoundland* or Labrador* or Nunavut* or NWT or "N.W.T" or "Northwest Territories" or Yukon* or Nunavik or Inuvialuit or Inuit* or Inuk or "First Nation" or "First Nations" or Metis or "Métis").ab,ti,kw,hw.
30. or/22-29
31. 10 and 21 and 30
32. limit 31 to yr="2008 -2021"

Ovid EBM Reviews - Cochrane Database of Systematic Reviews <2005 to July 14, 2021>

1. ("health equity" or "health services accessibility" or "right to health" or "healthcare disparities" or "health status disparities" or "social determinants of health" or "socioeconomic factors").kw.
2. ((health* or care) adj4 (Inequal* or unequal* or equal* or disparit* or gap? or oriented or accessibility or inaccessibility)).mp.
3. (equit* or inequit* or "social determinant*" or "right to health").mp.
4. ((socio* or social) adj2 (factor* or inequal* or unequal* or equal* or disparit* or gap? or accessibility or inaccessibility)).mp.
5. or/1-3
6. ("Neoplasms" or "Early Detection of Cancer" or "Cancer Survivors" or "Medical oncology" or "Psycho-oncology" or "Radiation oncology" or "Surgical oncology" or "Oncology Nursing" or "Oncology service, hospital" or "Cancer care facilities").kw.
7. (neoplas* or cancer* or tumor* or tumour* or carcinoma* or adenocarcinoma* or sarcoma* or leiomyosarcoma* or malignan* or oncolog*).mp.
8. 6 or 7
9. ("Indians, North American" or "Indigenous Canadians" or "Inuits").kw.
10. (canad* or "british columbia*" or Vancouver* or alberta* or Calgary or Calgari* or Edmonton* or saskatchewan* or manitoba* or ontari* or Ottawa* or Toronto* or quebec* or Montreal* or "nova scotia*" or "prince edward island*" or newfoundland* or labrador* or nunavut or nwt or "n.w.t" or "northwest territories" or yukon* or nunavik or inuvialuit or Inuit* or Inuk or "First Nation" or "First Nations" or Metis or "Métis").ab,kw,ti.
11. 9 or 10
12. 5 and 8 and 11
13. limit 12 to yr="2008-2021"

EBSCO CINAHL (inception to July 16, 2021)

S1. ((health* OR care) N3 (Inequal* OR unequal* OR equal* OR disparit* OR gap# OR oriented OR accessibility OR inaccessibility)) OR (equit* OR inequit* OR "social determinant*" OR "right to health*") OR ((socio* OR social) N1 (factor* OR Inequal* OR unequal* OR equal* OR disparit* OR gap# OR accessibility OR inaccessibility))

S2. (MH "Health services accessibility") OR (MH "Right to health") OR (MH "Healthcare disparities") OR (MH "Health status disparities") OR (MH "Social determinants of health") OR (MH "Socioeconomic factors")

S3. S1 OR S2

S4. (MH "Neoplasms+") OR (MH "Neoplasms by Site+") OR (MH "Neoplasms by Histologic Type+") OR (MH "Leukemia+") OR (MH "Lymphatic Vessel Tumors+") OR (MH "Lymphoma+") OR (MH "Neoplasms, Complex and Mixed+") OR (MH "Neoplasms, Connective and Soft Tissue+") OR (MH "Neoplasms, Germ Cell and Embryonal+") OR (MH "Neoplasms,Glandular and Epithelial+") OR (MH "Neoplasms, Gonadal Tissue+") OR (MH "Neoplasms, Nerve Tissue+") OR (MH "Neoplasms, Vascular Tissue+") OR (MH "Nevi and Melanomas+") OR (MH "Odontogenic Tumors+") OR (MH "Abdominal Neoplasms+") OR (MH "Bone Neoplasms+") OR (MH "Breast Neoplasms+") OR (MH "Digestive System

Neoplasms+") OR (MH "Endocrine Gland Neoplasms+") OR (MH "Eye Neoplasms+") OR(MH "Head and Neck Neoplasms+") OR (MH "Hematologic Neoplasms+") OR (MH "Nervous System Neoplasms+") OR (MH "Skin Neoplasms+") OR(MH "Sebaceous Gland Neoplasms+") OR (MH"Soft Tissue Neoplasms+") OR (MH "Thoracic Neoplasms+")OR (MH "Urogenital Neoplasms+") OR (MH "Heart Neoplasms+") OR (MH "Respiratory Tract Neoplasms+") OR (MH "Genital Neoplasms, Female+") OR (MH "Genital Neoplasms, Male+") OR (MH "Urologic Neoplasms+") OR (MH "Neoplasms, Hormone-Dependent+") OR (MH "Neoplasms, Multiple Primary+") OR (MH "Neoplasms, Radiation-Induced+") OR (MH "Multiple Endocrine Neoplasia+") OR (MH "Kidney Neoplasms+") OR (MH "Prostatic Neoplasms+") OR (MH "Ovarian Neoplasms+") OR (MH "Uterine Neoplasms+") OR (MH "Cervix Neoplasms+") OR (MH "Neoplasms, Second Primary") OR (MH "Leukemia, Lymphocytic+") OR (MH "Leukemia, Myeloid+") OR (MH "Leukemia, Myeloid, Acute+") OR (MH "Leukemia, Lymphocytic, Chronic+") OR (MH "Lymphoma, Non-Hodgkin's+") OR (MH "Plasmacytoma+") OR(MH "Lymphoma, B-Cell+") OR (MH "Lymphoma, T-Cell+") OR (MH "Lymphoma, T-Cell, Cutaneous+") OR (MH "Wilms' Tumor+") OR (MH "Lipoma+") OR (MH "Liposarcoma+") OR (MH "Neoplasms, Connective Tissue+") OR (MH "Neoplasms, Bone Tissue+") OR (MH "Neoplasms, Fibrous Tissue+") OR (MH "Osteosarcoma+") OR (MH "Neoplasms, Fibroepithelial+") OR (MH "Neoplasms, Muscle Tissue+") OR (MH "Myoma+") OR (MH "Myosarcoma+") OR (MH "Sarcoma+") OR (MH "Retroperitoneal Neoplasms+") OR (MH "Skull Neoplasms+") OR (MH "Jaw Neoplasms+") OR (MH "Biliary Tract Neoplasms+") OR (MH "Gastrointestinal Neoplasms+") OR (MH "Liver Neoplasms+") OR (MH "Pancreatic Neoplasms+") OR (MH "Adenoma, Islet Cell+") OR (MH "Carcinoma, Islet Cell+") OR (MH "Esophageal Neoplasms+") OR (MH "Intestinal Neoplasms+") OR (MH "Colorectal Neoplasms+") OR (MH"Adenomatous Polyposis Coli+") OR(MH "Colonic Neoplasms+") OR (MH "Rectal Neoplasms+") OR (MH "Anus Neoplasms+") OR (MH "Pituitary Neoplasms+") OR (MH "Adenoma, Pituitary+") OR (MH "Thyroid Neoplasms+") OR (MH "Retinal Neoplasms+") OR (MH"Facial Neoplasms+") OR (MH "Mouth Neoplasms+") OR (MH "Otorhinolaryngologic Neoplasms+") OR (MH "Salivary Gland Neoplasms+") OR (MH "Leukoplakia, Oral+") OR (MH "Oropharyngeal Neoplasms+") OR (MH "Nasopharyngeal Neoplasms+") OR (MH "Nose Neoplasms+") OR (MH "Pharyngeal Neoplasms+") OR (MH "Central Nervous System Neoplasms+") OR (MH "Neuroma, Acoustic+") OR (MH "Paraneoplastic Syndromes, Nervous System+") OR (MH "Peripheral Nervous System Neoplasms+") OR (MH "Brain Neoplasms+") OR (MH "Meningeal Neoplasms+") OR (MH "Infratentorial Neoplasms+") OR (MH "Supratentorial Neoplasms+") OR (MH "Hypothalamic Neoplasms+") OR (MH "Cranial Nerve Neoplasms+") OR (MH "Lung Neoplasms+") OR (MH "Pleural Neoplasms+") OR (MH "Early detection of cancer") OR (MH "Cancer survivors") OR (MH "Oncology+") OR(MH "Oncologic care+") OR (MH "Oncology care units") OR (MH "Cancer care facilities")

S5. neoplas* OR cancer* OR tumor* OR tumour* OR carcinoma* OR adenocarcinoma* OR sarcoma* OR leiomyosarcoma* OR malignan* OR oncolog*

S6. S4 OR S5

S7. AB (Canad* OR "British Columbia*" OR Vancouver* OR Alberta* OR Calgary OR Calgari* OR Edmonton* OR Saskatchewan* OR Manitoba* OR Ontari* OR Ottawa* OR Toronto* OR Quebec* OR Montreal* OR "Nova Scotia*" OR "Prince Edward Island*" OR Newfoundland* OR Labrador* OR Nunavut* OR NWT OR "N.W.T" OR "Northwest

Territories" OR Yukon* OR Nunavik OR Inuvialuit OR Inuit* OR Inuk OR "First Nation" OR "First Nations" OR Metis OR "Métis") OR TI (Canad* OR "British Columbia*" OR Vancouver* OR Alberta* OR Calgary OR Calgari* OR Edmonton* OR Saskatchewan* OR Manitoba* OR Ontari* OR Ottawa* OR Toronto* OR Quebec* OR Montreal* OR "Nova Scotia*" OR "Prince Edward Island*" OR Newfoundland* OR Labrador* OR Nunavut* OR NWT OR "N.W.T" OR "Northwest Territories" OR Yukon* OR Nunavik OR Inuvialuit OR Inuit* OR Inuk OR "First Nation" OR "First Nations" OR Metis OR "Métis") OR MW (Canad* OR "British Columbia*" OR Vancouver* OR Alberta* OR Calgary OR Calgari* OR Edmonton* OR Saskatchewan* OR Manitoba* OR Ontari* OR Ottawa* OR Toronto* OR Quebec* OR Montreal* OR "Nova Scotia*" OR "Prince Edward Island*" OR Newfoundland* OR Labrador* OR Nunavut* OR NWT OR "N.W.T" OR "Northwest Territories" OR Yukon* OR Nunavik OR Inuvialuit OR Inuit* OR Inuk OR "First Nation" OR "First Nations" OR Metis OR "Métis")

S8. MW (MH "Aboriginal Canadians") OR (MH "First Nations of Canada") OR (MH "Inuit")

S9. S7 OR S8

S10. S3 AND S6 AND S9 - Limiters - PublishedDate: 20080101-20211231

EBSCO APA PSYCINFO (inception to July 16, 2021)

S1. ((health* OR care) N3 (Inequal* OR unequal* OR equal* OR disparit* OR gap# OR oriented OR accessibility OR inaccessibility)) OR (equit* OR inequit* OR "social determinant*" OR "right to health*") OR ((socio* OR social) N1 (factor* OR Inequal* OR unequal* OR equal* OR disparit* OR gap# OR accessibility OR inaccessibility))

S2. DE "Equity" OR DE "Health Care Access" OR DE "Right to Treatment" OR DE "Health Disparities" OR DE "Socioeconomic Factors"

S3. S1 OR S2

S4. DE "Neoplasms" OR DE "Breast Neoplasms" OR DE "Endocrine Neoplasms" OR DE "Leukemias" OR DE "Melanoma" OR DE "Metastasis" OR DE "Terminal Cancer" OR DE "Nervous System Neoplasms" OR DE "Brain Neoplasms" OR DE "Glioma" OR DE "Oncology"

S5. neoplas* OR cancer* OR tumor* OR tumour* OR carcinoma* OR adenocarcinoma* OR sarcoma* OR leiomyosarcoma* OR malignan* OR oncolog*

S6. S4 OR S5

S7. DE "American Indians" OR DE "Inuit"

S8. AB (Canad* OR "British Columbia*" OR Vancouver* OR Alberta* OR Calgary OR Calgari* OR Edmonton* OR Saskatchewan* OR Manitoba* OR Ontari* OR Ottawa* OR Toronto* OR Quebec* OR Montreal* OR "Nova Scotia*" OR "Prince Edward Island*" OR Newfoundland* OR Labrador* OR Nunavut* OR NWT OR "N.W.T" OR "Northwest Territories" OR Yukon* OR Nunavik OR Inuvialuit OR Inuit* OR Inuk OR "First Nation" OR "First Nations" OR Metis OR "Métis") OR TI (Canad* OR "British Columbia*" OR Vancouver* OR Alberta* OR Calgary OR Calgari* OR Edmonton* OR Saskatchewan* OR Manitoba* OR Ontari* OR Ottawa* OR Toronto* OR Quebec* OR Montreal* OR "Nova Scotia*" OR "Prince Edward Island*" OR Newfoundland* OR Labrador* OR Nunavut* OR NWT OR "N.W.T" OR

"Northwest Territories" OR Yukon* OR Nunavik OR Inuvialuit OR Inuit* OR Inuk OR "First Nation" OR "First Nations" OR Metis OR "Métis") OR KW (Canad* OR "British Columbia*" OR Vancouver* OR Alberta* OR Calgary OR Calgari* OR Edmonton* OR Saskatchewan* OR Manitoba* OR Ontari* OR Ottawa* OR Toronto* OR Quebec* OR Montreal* OR "Nova Scotia*" OR "Prince Edward Island*" OR Newfoundland* OR Labrador* OR Nunavut* OR NWT OR "N.W.T" OR "Northwest Territories" OR Yukon* OR Nunavik OR Inuvialuit OR Inuit* OR Inuk OR "First Nation" OR "First Nations" OR Metis OR "Métis")

S9. S7 OR S8

S10. S3 AND S6 AND S9 - Limiters - Published Date: 20080101- 20211231

Grey Literature Search Strategy:

**Cancer websites**	**URL**
Alberta Health Services (includes Cancer Care Alberta)	https://www.albertahealthservices.ca
BC Cancer, Provincial Health Services Authority	http://www.bccancer.bc.ca
Canadian Cancer Society	https://cancer.ca/en
Canadian Partnership Against Cancer	https://www.partnershipagainstcancer.ca
Cancer Care Ontario, Ontario Health	https://www.cancercareontario.ca/en
CancerCare Manitoba	https://www.cancercare.mb.ca
Children's Oncology Group	https://childrensoncologygroup.org/
Eastern Health (includes Eastern Health Cancer Care)	https://www.easternhealth.ca/<br>https://cancercare.easternhealth.ca/
Health Department, New Brunswick Government (includes New Brunswick Cancer Network)	https://www2.gnb.ca/content/gnb/en/departments/health.html
Health PEI Staff Resources Centre	https://src.healthpei.ca/
Quebec Ministry of Health and Social Services/ Ministère de la Santé et des Services Sociaux website	https://www.msss.gouv.qc.ca/
Nova Scotia Health Authority (includes Nova Scotia Health Authority Cancer Care Program and Nova Scotia Breast Screening Program)	http://www.nshealth.ca/<br>https://breastscreening.nshealth.ca/
Prince Edward Island Government (includes PEI Cancer Treatment Centre)	https://www.princeedwardisland.ca/
Saskatchewan Cancer Agency	http://www.saskcancer.ca/

The Canadian Cancer Research Alliance	https://www.ccra-acrc.ca/

Keywords searched on cancer websites:

- Health equity
- Health care equity
- Healthcare equity
- Equity
- Equities
- Equitable
- Inequity
- Inequities
- Inequitable
- Disparity
- Disparities
- Equality
- Equalities
- Equal
- Equally
- Inequality
- Inequalities
- Unequal
- Unequally

Keywords searched in French on the Quebec Ministry of Health and Social Services/ Ministère de la Santé et des Services Sociaux website:

- équité en santé = health equity
- soins équitables = equitable care
- un accès égal aux soins de santé = equal access to healthcare
- disparités dans la prise en charge du cancer = disparities in cancer care

**Non-Oncology websites**	**URL**
Alberta Health Services (includes Cancer Care Alberta)	https://www.albertahealthservices.ca
BC Children's Hospital, Provincial Health Services Authority	http://www.bcchildrens.ca
Canadian Institute for Health Information	https://www.cihi.ca/en
Canadian Institutes of Health Research	https://cihr-irsc.gc.ca/e/193.html
Centre for Health Economics and Policy Analysis	https://chepa.mcmaster.ca/
Child & Family Research Institute	https://www.cfri.ca/
Eastern Health	https://www.easternhealth.ca/
Health Canada	https://www.canada.ca/en/health-canada.html
Health Department, New Brunswick Government (includes New Brunswick Cancer Network)	https://www2.gnb.ca/content/gnb/en/departments/health.html
Health PEI Staff Resources Centre	https://src.healthpei.ca/
Indigenous Studies Portal - University of	https://iportal.usask.ca/

Saskatchewan	
Quebec Ministry of Health and Social Services/ Ministère de la Santé et des Services Sociaux website.	https://www.msss.gouv.qc.ca/
National Center for Complementary and Integrative Health	https://www.nccih.ca/en/
National Collaborating Centre for Determinants of Health	https://nccdh.ca/
National Collaborating Centre for Environmental Health	https://www.ncceh.ca/
National Collaborating Centre for Healthy Public Policy	https://ccnpps-ncchpp.ca/
National Collaborating Centre for Infectious Diseases	https://nccid.ca/
National Collaborating Centre for Methods and Tools	https://www.nccmt.ca/
Native Health Database	https://hslic-nhd.health.unm.edu/<br>https://nativehealthdatabase.net/
Nova Scotia Health Authority (includes Nova Scotia Health Authority Cancer Care Program)	http://www.nshealth.ca/
Prince Edward Island Government (includes PEI Cancer Treatment Centre)	https://www.princeedwardisland.ca/
SSRN (Social Science Research Network)	https://www.ssrn.com/index.cfm/en/

Keywords searched for non-oncology sources:

- "Health equity" Cancer
- "Health care equity" Cancer
- "Healthcare equity" Cancer
- "Equity" Cancer
- "Equities" Cancer
- "Equitable" Cancer
- "Inequity" Cancer
- "Inequities" Cancer
- "Inequitable" Cancer
- "Disparity" Cancer
- "Disparities" Cancer
- "Equality" Cancer
- "Equalities" Cancer
- "Equal" Cancer
- "Equally" Cancer
- "Inequality" Cancer
- "Inequalities" Cancer
- "Unequal" Cancer

- "Unequally" Cancer
- "Health equity" Oncology
- "Health care equity" Oncology
- "Healthcare equity" Oncology
- "Equity" Oncology
- "Equities" Oncology
- "Equitable" Oncology
- "Inequity" Oncology
- "Inequities" Oncology
- "Inequitable" Oncology
- "Disparity" Oncology
- "Disparities" Oncology
- "Equality" Oncology
- "Equalities" Oncology
- "Equal" Oncology
- "Equally" Oncology
- "Inequality" Oncology
- "Inequalities" Oncology
- "Unequal" Oncology
- "Unequally" Oncology

Keywords searched in Google Advanced Search (https://www.google.ca/advanced_search) for Internet-Wide Search:

- Cancer equity
- Cancer disparities

Limiters:

- File Type: PDF
- Region: Canada
