# Supplementary material for: Health and healthcare equity within the Canadian cancer care sector: a rapid scoping review
Source: Int J Equity Health. 2023 Jan 28;22:20. doi: 10.1186/s12939-023-01829-2 (PMC9883825; doi:10.1186/s12939-023-01829-2)
Supplement: Supplementary file 2 — Additional file 2. Complete list of documents included for analysis. [file 12939_2023_1829_MOESM2_ESM.pdf]

ADDITIONAL FILE #2:
Complete List of Documents Included for Analysis

Ahmed S, Shahid RK. Disparity in cancer care: A Canadian perspective. *Curr Oncol*. 2012;19(6):376-382. doi:10.3747/co.19.1177

Ahmed S, Shahid RK, Episkenew JA. Disparity in cancer prevention and screening in Aboriginal populations: Recommendations for action. *Curr Oncol*. 2015;22(6):417-426. doi:10.3747/co.22.2599

Alberta Health Services. *Palliative and End of Life Care: Alberta Provincial Framework*. 2014. https://www.albertahealthservices.ca/assets/info/seniors/if-sen-provincial-palliative-end-of-life-care-framework.pdf.

Algu K. Denied the right to comfort: Racial inequities in palliative care provision. *EClinicalMedicine*. 2021;34:100833. doi:10.1016/j.eclinm.2021.100833

BC Patient Safety & Quality Council. *Journey Mapping in Cancer Care: Patient and Provider Experiences in Receiving and Delivering Cancer Care in British Columbia*. Vancouver; 2019. https://bcpsqc.ca/wp-content/uploads/2019/01/Journey-Mapping-Cancer-interactive.pdf.

Bell JAH, Escaf M, Schmilovich Z, Costello J, Buchman DZ, Messner HA. First ready, first to go: Ethical priority-setting of allogeneic stem cell transplant at a major cancer centre. *Healthc Policy*. 2020;15(3):103-115. doi:10.12927/HCPOL.2020.26127

Bentley C, Peacock S, Abelson J, et al. Addressing the affordability of cancer drugs: Using deliberative public engagement to inform health policy. *Heal Res Policy Syst*. 2019;17(1):1-10. doi:10.1186/s12961-019-0411-8

Blair A, Gauvin L, Strumpf EC, Datta GD. Impact of organized colorectal cancer screening programs on screening uptake and screening inequalities: A study of systematic- and patient-reliant programs in Canada. *J Cancer Policy*. 2020;24(April):100229. doi:10.1016/j.jcpo.2020.100229

Canadian Cancer Research Alliance. *Cancer Prevention Research in Canada: A Strategic Framework for Collaborative Action*. Toronto; 2012. https://www.ccra-acrc.ca/wp-content/uploads/2020/08/Prev_Res_Strat_Framework-2.pdf.

Canadian Cancer Research Alliance. *Pan-Canadian Framework for Palliative and End-of-Life Care Resaerch*. Toronto; 2017. https://www.ccra-acrc.ca/wp-content/uploads/2020/08/PEOLC_Framework_2017_EN.pdf.

Canadian Cancer Research Alliance. *Recommendations to Guide Implementation Planning for Cancer Health Services and Policy Research in Canada*. Toronto; 2021. https://www.ccra-acrc.ca/wp-content/uploads/2021/07/HSPR_Recommendations_EN.pdf.

Canadian Cancer Society. *Canadian Cancer Statistics: A 2020 Special Report on Lung Cancer*. Toronto; 2020. https://cdn.cancer.ca/-/media/files/cancer-information/resources/publications/2020-canadian-cancer-statistics-special-report/2020-canadian-cancer-statistics-special-report-en.pdf?rev=15c66a0b05f5479e935b48035c70dca3&hash=3D51B0D0FB5C3F7E659F896D66495CE8&_.

Canadian Cancer Society. COVID-19 Response. https://cancer.ca/en/get-involved/advocacy/what-we-are-doing/covid-19-response. Published 2021. Accessed January 31, 2022.

Canadian Cancer Society. Access to cancer drugs and diagnostics. https://cancer.ca/en/get-involved/advocacy/what-we-are-doing/drug-access. Published 2021. Accessed January 31, 2022.

Canadian Institutes of Health Research. Institute of Cancer Research: Strategic Plan 2015-2020. https://cihr-irsc.gc.ca/e/49894.html. Published 2016. Accessed January 31, 2022.

Canadian Partnership Against Cancer. *Action Plan for the Elimination of Cervical Cancer in Canada 2020-2030*. Toronto; 2020. https://s22438.pcdn.co/wp-content/uploads/2020/11/Elimination-cervical-cancer-action-plan-EN.pdf.

Canadian Partnership Against Cancer. *Promoting Equity and Diversity in Cancer Care Settings*. Toronto; 2009. https://dev.partnershipagainstcancer.ca/wp-content/uploads/2018/12/Promoting-Equity-Diversity-EN.pdf.

Canadian Partnership Against Cancer. *Management of Cancer Screening Services during the COVID-19 Pandemic: Guidance Document*. Toronto; 2020. https://www.partnershipagainstcancer.ca/topics/cancer-screening-covid-19/.

Canadian Partnership Against Cancer. *Cervical Cancer: HPV Primary Screening and Abnormal Screen Follow-up Environmental Scan*. Toronto; 2021. https://s22457.pcdn.co/wp-content/uploads/2021/03/HPV-primary-screening-escan-EN.pdf.

Canadian Partnership Against Cancer. *Lung Cancer and Equity: A Focus on Income and Geography*. Toronto; 2020. https://www.partnershipagainstcancer.ca/wp-content/uploads/2020/11/Lung-cancer-and-equity-report-EN.pdf.

Canadian Partnership Against Cancer. *Equity-Focused Interventions to Increase Colorectal Cancer Screening: Program Pack*. Toronto; 2021. https://www.partnershipagainstcancer.ca/topics/equity-colorectal-cancer-screening/.

Canadian Partnership Against Cancer. *Canadian Strategy for Cancer Control*. Toronto, ON; 2019. https://s22457.pcdn.co/wp-content/uploads/2019/06/Canadian-Strategy-Cancer-Control-2019-2029-EN.pdf.

Canadian Partnership Against Cancer. *Annual Report 2019/20: Doing What Can Only Be Done*

Together. Toronto; 2020. https://s22457.pcdn.co/wp-content/uploads/2020/10/Annual-report-2019-2020-EN.pdf.

Canadian Partnership Against Cancer. *Cancer System Performace: 2017 Report*. Toronto; 2017. https://s22457.pcdn.co/wp-content/uploads/2019/12/2017_cancer_system_performance_report_EN.pdf.

Canadian Partnership Against Cancer. Examining Disparities in Cancer Control system performance special focus report. 2014;(February):88. https://www.partnershipagainstcancer.ca/topics/disparities-in-cancer-control/.

CancerCare Manitoba. *2016-2021 Manitoba Cancer Plan*. Winnipg; 2016. https://www.cancercare.mb.ca/export/sites/default/About-Us/.galleries/files/corporate-publications/Manitoba_Cancer_Plan_2016-2021.pdf

CancerCare Manitoba. Underserved Populations Program. https://www.cancercare.mb.ca/Patient-Family/underserved-populations. Accessed January 31, 2022.

CancerCare Manitoba. Chapter 5: Facilitating an Inclusive Environment. In: *Cervical Cancer Screening Learning Module for Healthcare Providers*. Winnipeg: CancerCare Manitoba; 2009. https://www.cancercare.mb.ca/export/sites/default/screening/.galleries/files/cervixcheck-ptlm/x-ptlm-ch5.pdf.

CancerCare Manitoba. *Roadmap to Cancer Control for Manitoba: Setting Priorities, Working Together and Achieving the Best Results*. Winnipeg, Canada; 2020. https://www.cancercare.mb.ca/export/sites/default/.galleries/files/Roadmap-to-Cancer-Control-MB.pdf

Cancer Care Ontario. *First Nations, Inuit, Metis and Urban Indigenous Cancer Strategy*. Toronto; 2019. https://www.cancercareontario.ca/en/cancer-care-ontario/programs/aboriginal-programs/indigenous-cancer-strategy.

Cancer Care Ontario. *Prevention System Quality Index: Health Equity*. Toronto; 2018. https://www.cancercareontario.ca/sites/ccocancercare/files/assets/PSQI2018-FullReport.pdf.

Cancer Care Ontario. Ontario Cancer Plan 5: 2019-2023. https://www.cancercareontario.ca/sites/ccocancercare/files/assets/CancerSystemPlanSummary-6-19.pdf. Published 2019; accessed July 22, 2021.

Cancer Care Ontario. *Central East Regional Cancer Program 2019-2023 Refreshed Strategic Plan*. Oshawa, Ontario; 2019. https://www.lakeridgehealth.on.ca/en/central-east-regional-cancer-program/resources/CE-RCP-Strategic-Plan-2019-23.pdf.

Cancer Care Ontario. *Erie St. Clair Regional Cancer Program Strategic Plan 2016-2019*. Windsor, Ontario; 2016.

https://www.wrh.on.ca/uploads/Common/Cancer_Program_Strategic_Plan_2016_2019.pdf.

Cancer Care Ontario. *Northeast Aboriginal Cancer Plan 2015-2019*. 2015. https://www.cancercare.on.ca/common/pages/UserFile.aspx?fileId=345455.

Cancer Care Ontario. *South East Regional Cancer Program Regional Cancer Plan 2016-2019*. Kingston, Ontario; 2016. http://cancercaresoutheast.ca/sites/default/files/documents/sercp_stratplan_2016-2019.pdf.

Cancer Control Alberta. *Supportive Care Framework Report*. Edmonton; 2016. https://www.albertahealthservices.ca/assets/info/hp/cancer/if-hp-cancer-supportive-care-framework-report.pdf.

Chan J, Friborg J, Zubizarreta E, et al. Examining geographic accessibility to radiotherapy in Canada and Greenland for indigenous populations: Measuring inequities to inform solutions. *Radiother Oncol*. 2020;146. doi:10.1016/j.radonc.2020.01.023

Cobigo V, Ouellette-Kuntz H, Balogh R, Leung F, Lin E, Lunsky Y. Are cervical and breast cancer screening programmes equitable? The case of women with intellectual and developmental disabilities. *J Intellect Disabil Res*. 2013;57(5):478-488. doi:10.1111/jir.12035

Erdman JN. Health equity, HPV and the cervical cancer vaccine. *Health Law J*. 2008;Special Ed:127-143.

Feldman J, Davie S, Kiran T. Measuring and improving cervical, breast, and colorectal cancer screening rates in a multi-site urban practice in Toronto, Canada. *BMJ Qual Improv Reports*. 2017;6(1):u213991.w5531. doi:10.1136/bmjquality.u213991.w5531

Fernandez C, Fraser GAM, Freeman C, et al. Principles and Recommendations for the Provision of Healthcare in Canada to Adolescent and Young Adult–Aged Cancer Patients and Survivors. *J Adolesc Young Adult Oncol*. 2011;1(1):53-59. doi:10.1089/jayao.2010.0008

Glicksman RM, Wong A, Wang J, et al. The Capital Investment Strategy for Radiation therapy in Ontario: A Framework to Ensure Access to Radiation Therapy. *Adv Radiat Oncol*. 2020;5(3):318-324. doi:10.1016/j.adro.2019.12.004

Gould J, Sinding C, Mitchell TL, et al. "below their notice": Exploring women's subjective experiences of cancer system Exclusion. *J Cancer Educ*. 2009;24(4):308-314. doi:10.1080/08858190902997324

Hammond C, Thomas R, Gifford W, et al. Cycles of silence: First Nations women overcoming social and historical barriers in supportive cancer care. *Psychooncology*. 2017;26(2):191-198. doi:10.1002/pon.4335

Henderson RI, Shea-Budgell M, Healy C, et al. First nations people's perspectives on barriers and supports for enhancing HPV vaccination: Foundations for sustainable, community-driven

strategies. *Gynecol Oncol*. 2018;149(1):93-100. doi:10.1016/j.ygyno.2017.12.024

Honein-AbouHaidar GN, Baxter NN, Moineddin R, Urbach DR, Rabeneck L, Bierman AS. Trends and inequities in colorectal cancer screening participation in Ontario, Canada, 2005-2011. *Cancer Epidemiol*. 2013;37(6):946-956. doi:10.1016/j.canep.2013.04.007

Horrill TC, Lavoie JG, Martin D, Schultz ASH. Places & spaces: A critical analysis of cancer disparities and access to cancer care among First Nations Peoples in Canada. *Witn Can J Crit Nurs Discourse*. 2020;2(2):104-123. doi:doi.10.25071/2291-5796.62

Horrill TC, Linton J, Lavoie JG, Martin D, Wiens A, Schultz ASH. Access to cancer care among Indigenous peoples in Canada: A scoping review. *Soc Sci Med*. 2019;238. doi:10.1016/j.socscimed.2019.112495

Inuit Tapirit Kanatami. *Inuit & Cancer: Discussion Paper*. Ottawa; 2008. https://www.itk.ca/wp-content/uploads/2016/07/Final-Inuit-and-Cancer-Discussion-Paper-October-2008.pdf.

Kewayosh A, Marrett L, Aslam U, et al. Improving health equity for First Nations, Inuit and Métis people: Ontario's Aboriginal Cancer Strategy II. *Healthc Q*. 2015;17:33-40. doi:10.12927/hcq.2014.24007

Legere LE, MacDonnell JA. Meaningful support for lesbian and bisexual women navigating reproductive cancer care in Canada: An exploratory study. *J Res Nurs*. 2016;21(3):163-174. doi:10.1177/1744987116640582

Lofters A, Kiran T. *Improving Cancer Screening Rates in Your Practice and Reducing Related Disparities*. Toronto; 2019. https://maphealth.ca/wp-content/uploads/2019/11/MAP-toolkit-for-health-care-professionals-Improving-cancer-screening-rates-in-your-practice-and-reducing-related-disparities.pdf.

Lofters A, Virani T, Grewal G, Lobb R. Using knowledge exchange to build and sustain community support to reduce cancer screening inequities. *Prog Community Heal Partnerships Res Educ Action*. 2015;9(3):379-387. doi:10.1353/cpr.2015.0064

Lung Cancer Canada. *2015 Faces of Lung Cancer Report*. Toronto; 2015. https://www.lungcancercanada.ca/LungCancerCanada/media/Documents/The-Faces-of-Lung-Cancer-2015.pdf.

Lung Health Foundation, Lung Cancer Canada. *Start Asking the Right Questions about Lung Cancer: A Roadmap for Lasting Change*. Toronto; 2020. https://thewrongquestion.ca/wp-content/uploads/2020/10/lhf_lungcancer_antistigma_report_eng_rgb-1.pdf.

Maar M, Burchell A, Little J, et al. A qualitative study of provider perspectives of structural barriers to cervical cancer screening among first nations women. *Women's Heal Issues*. 2013;23(5):e319-e325. doi:10.1016/j.whi.2013.06.005

Maddison AR, Asada Y, Urquhart R. Inequity in access to cancer care: A review of the Canadian literature. *Cancer Causes Control*. 2011;22(3):359-366. doi:10.1007/s10552-010-9722-3

Maddison AR, Asada Y, Urquhart R, Johnston G, Burge F, Porter G. Inequity in access to guideline-recommended colorectal cancer treatment in Nova Scotia, Canada. *Healthc Policy*. 2012;8(2):71-87. doi:10.12927/hcpol.2012.23131

Miller PA, Sinding C, McGillicuddy P, et al. Disparities in cancer care: Perspectives from the front line. *Palliat Support Care*. 2014;12(3):175-181. doi:10.1017/S147895151200106X

National Collaborating Centre for Methods and Tools. *The Evidence-Informed Decision-Making Casebook: Issue One*. Hamilton; N.D. https://www.nccmt.ca/uploads/media/media/0001/02/878448c75322064d1400d6413fd50962599713f0.pdf.

Nova Scotia Cancer Care Program. *Yarmouth Area Cancer Services Review*. Halifax, Canada; 2018. https://www.nshealth.ca/sites/nshealth.ca/files/yarmouth_area_cancer_services_review.pdf.

Purificacion SJ, French JG, D'Agincourt-Canning L. Inequities in access to cancer care in Canada: An ethical perspective. *Healthc Manag Forum*. 2015;28(6):265-269. doi:10.1177/0840470415599136

Raynault MF, Féthière C, Côté D. Social inequalities in breast cancer screening: evaluating written communications with immigrant Haitian women in Montreal. *Int J Equity Health*. 2020;19:209. doi:10.1186/s12939-020-01322-0

Sayani A. Health equity in national cancer control plans: An analysis of the ontario cancer plan. *Int J Heal Policy Manag*. 2019;8(9):550-556. doi:10.15171/ijhpm.2019.40

Sayani A. Inequities in genetic testing for hereditary breast cancer: implications for public health practice. *J Community Genet*. 2019;10(1):35-39. doi:10.1007/s12687-018-0370-8

Sayani A, Vahabi M, O'Brien MA, et al. Advancing health equity in cancer care: The lived experiences of poverty and access to lung cancer screening. *PLoS One*. 2021;16(5 May):1-16. doi:10.1371/journal.pone.0251264

Sayani A, Vahabi M, O'Brien MA, et al. Perspectives of family physicians towards access to lung cancer screening for individuals living with low income: A qualitative study. *BMC Fam Pract*. 2021;22(1):1-9. doi:10.1186/s12875-020-01354-z

Scime S. Inequities in cancer care among transgender people: Recommendations for change. *Can Oncol Nurs J*. 2019;29(2):87-91. doi:10.5737/236880762928791

Sinding C, Miller P, Hudak P, Keller-Olaman S, Sussman J. Of time and troubles: Patient involvement and the production of health care disparities. *Health*. 2012;16(4):400-417.

doi:10.1177/1363459311416833

Sinding C, Watt L, Miller P, et al. Stigmas and Silos: Social Workers' Accounts of Care for People With Serious Mental Illness and Cancer. *Soc Work Ment Health*. 2013;11(3):288-309. doi:10.1080/15332985.2012.758075

So WK, Chan RJ, Truant T, Trevatt P, Bialous SA, Barton-Burke M. Global perspectives on cancer health disparities: Impact, utility, and implications for cancer nursing. *Asia-Pacific J Oncol Nurs*. 2016;3(4):316-323. doi:10.4103/2347-5625.195885

Soo J, French J, McGahan CE, Duncan G, Lengoc S. A retrospective study on accessibility of palliative radiation therapy in the management of prostate cancer in British Columbia. *J Radiother Pract*. 2011;10(3):159-172. doi:10.1017/S1460396910000348

Sorin M, Franco EL, Quesnel-Vallée A. Inter-and intraprovincial inequities in public coverage of cancer drug programs across Canada: A plea for the establishment of a pan-canadian pharmacare program. *Curr Oncol*. 2019;26(4):266-269. doi:10.3747/co.26.4867

South Riverdale Community Health Centre, Mount Sinai Hospital, Toronto Public Health. *Engaging Seldom or Never Screened Women in Cancer Screening: A Compendium of Pan-Canadian Best and Promising Practices*. Toronto; 2010. https://www.mountsinai.on.ca/care/mkbc/resources-2/engagingwomenincancerscreening-compressed.pdf.

Thorne S, Truant T. Will designated patient navigators fix the problem? Oncology nursing in transition. *Can Oncol Nurs J*. 2010;20(3):116-128. doi:10.5737/1181912x203116121

Truant TL, Fitch MI, O'Leary C, Stewart J. Global perspectives on cancer survivorship: From lost in transition to leading into the future. *Can Oncol Nurs J*. 2017;27(3):287-294. http://www.ncbi.nlm.nih.gov/pubmed/31148806%0Ahttp://www.pubmedcentral.nih.gov/articlerender.fcgi?artid=PMC6516397.

Truant TLO, Lambert LK, Thorne S. Barriers to equity in cancer survivorship care: Perspectives of cancer survivors and system stakeholders. *Glob Qual Nurs Res*. 2021;8. doi:10.1177/23333936211006703

Truant T, Varcoe C, Gotay C, Thorne S. Toward equitably high-quality cancer survivorship care. *Can Oncol Nurs J*. 2019;29(3):156-162. doi:10.5737/23688076293156162

Vahabi M, Lofters A, Kumar M, Glazier RH. Breast cancer screening disparities among immigrant women by world region of origin: a population-based study in Ontario, Canada. *Cancer Med*. 2016;5(7):1670-1686. doi:10.1002/cam4.700

Varcoe C, Sinding C, Fitch M. Health disparities in cancer care: Exploring Canadian, American and international perspectives. *Can Oncol Nurs J*. 2015;25(1):73-81.

Yee EK, Coburn NG, Zuk V, et al. Geographic impact on access to care and survival for non-curative esophagogastric cancer: a population-based study. *Gastric Cancer*. 2021;24(4):790-799. doi:10.1007/s10120-021-01157-w

Zehbe I, Magajna B. *Anishinaabek Cervical Cancer Screening Study: Community Update Report*. Thunder Bay; 2015. http://www.accssfn.com/uploads/1/4/1/6/14167096/accss_report_cihr.pdf
