# Supplementary material for: Health and healthcare equity within the Canadian cancer care sector: a rapid scoping review
Source: Int J Equity Health. 2023 Jan 28;22:20. doi: 10.1186/s12939-023-01829-2 (PMC9883825; doi:10.1186/s12939-023-01829-2)
Supplement: Supplementary file 3 — Additional file 3. Variables for data charting. [file 12939_2023_1829_MOESM3_ESM.pdf]

ADDITIONAL FILE #3:
Variables for Data Charting

Category	Variable
**Publication characteristics**	Author or organization<br>Title<br>Year of publication<br>Type of publication/document<br>Source of publication/document
**Health equity goal(s)**	-What is the stated health or health care equity goal, if any?<br>-Is there acknowledgment of the causes of inequities? Describe<br>-At what level is the goal targeted? (i.e., individuals, health system, structural)
**Population**	Is the health equity goal specific to a population or group? Describe
**Health equity action**	-What actions (interventions, policies, strategies) are described to address the health equity goal?<br>-Do the actions align with the stated health equity goal and/or stated causes of inequities?<br>-Who is responsible/accountable for taking action on the health equity goal?
**Context**	What is the context of the health equity goal and/or action? (i.e., organizational, community, regional, provincial, national)
**Outcomes**	Is progress on the health equity goal being measured? If so, how?
